# Combination of MLo-1508 with sunitinib for the experimental treatment of papillary renal cell carcinoma

**DOI:** 10.3389/fonc.2025.1399956

**Published:** 2025-03-24

**Authors:** Ângela Marques-Magalhães, Filipa Moreira-Silva, Inês Graça, Paula C. Dias, Margareta P. Correia, Maria Ana Alzamora, Rui Henrique, Marie Lopez, Paola B. Arimondo, Vera Miranda-Gonçalves, Carmen Jerónimo

**Affiliations:** ^1^ Cancer Biology and Epigenetics Group, Research Center of IPO Porto (CI-IPOP)/CI-IPOP@RISE (Health Research Network), Portuguese Oncology Institute of Porto (IPO Porto)/Porto Comprehensive Cancer Center (Porto.CCC), Porto, Portugal; ^2^ Department of Pathology, Portuguese Oncology Institute of Porto (IPO Porto), Porto, Portugal; ^3^ Department of Pathology and Molecular Immunology, School of Medicine and Biomedical Sciences of the University of Porto (ICBAS-UP), Porto, Portugal; ^4^ Institut des Biomolécules Max Mousseron (IBMM), UMR 5247, CNRS-Université de Montpellier-ENSCM, Montpellier, France; ^5^ Epigenetic Chemical Biology, Institut Pasteur, UMR 3523CNRS, Paris, France

**Keywords:** renal cell carcinoma, epigenetics, DNA methylation, MLo-1508, sunitinib

## Abstract

Renal cell carcinoma (RCC) is the 14^th^ most incident cancer worldwide, and no curative therapeutic options are available for advanced and metastatic disease. Hence, new treatment alternatives are urgently needed to tackle disease management and drug resistance. Herein, we explored the use of MLo-1508 as an anti-tumoral agent in RCC and further assessed its combination with sunitinib for the treatment of papillary RCC. For that, different RCC cell lines were treated with both drugs, alone or in combination, and different phenotypic assays were performed. Moreover, global DNA methylation levels and specific DNMT3a activity were measured, and gene-specific CpG methylation and transcript levels were quantified after treatment. Finally, the combinatory potential of MLo-1508 and sunitinib were asses both in vitro and in vivo using the ACHN cell line. We found that MLo-1508 significantly decreased RCC cell viability while inducing apoptosis in a dose-dependent manner without cytotoxicity for non-malignant cells. Moreover, the treatment induced morphometric alterations and DNA damage in all RCC cell lines. MLo-1508 decreased *DNMT1* and *DNMT3A* transcript levels in 786-O and ACHN cells, inhibited DNMT3A activity, and reduced the global DNA methylation content of ACHN cells. When combined with sunitinib, a reduction in ACHN cell viability, as well as cell cycle arrest at G2/M was observed. Importantly, MLo-1508 decreased the sunitinib effective anti-tumoral concentration against ACHN cell viability. In an *in vivo* ACHN CAM model, the combination induced cell necrosis. Thus, MLo-1508 might improve sensitivity to sunitinib treatment by decreasing the required concentration and delaying resistance acquisition.

## 1 Introduction

Renal cell carcinoma (RCC) is the 14th most incident and the 15th most deadly cancer type worldwide (1). RCC encompasses heterogeneous subtypes that are vastly diverse at the biological and clinical level (2). The most common RCC subtype is clear cell RCC (ccRCC, 75% of the cases), followed by papillary (pRCC, 10-15% of the cases) and chromophobe (chRCC, 5% of the cases) (3, 4). Nowadays, roughly 30% of the RCC diagnosed cases present locally advanced or metastatic disease (5), and despite the observed disease control after treatment with tyrosine kinase inhibitors (TKI) (6), patients eventually develop resistance (7), highlighting the demand for new and effective therapeutic approaches and regimes.

Epigenetics, as a hallmark of cancer (8), has been largely associated with disease progression (9) and, in RCC, DNA hypermethylation of the promoter regions of tumor suppressor genes has been widely described (10–13). DNA methylation, written by DNA methyltransferases (DNMTs) is a reversible process (11) that can be regulated by the use of DNMT inhibitors (14, 15). Indeed, both the Food and Drug Administration (FDA) and the European Medicines Agency (EMA) have already approved two nucleoside DNMT inhibitors for clinical use (16, 17). However, due to their reduced efficacy in solid tumors (18), new, effective, and less toxic alternatives are being explored.

Natural compounds, particularly flavonoids, and their derivatives have been studied for their anti-neoplastic effects, based on the inhibition of DNMT activity (19, 20). Additionally, these compounds were shown to induce apoptosis and inhibit proliferation, migration, and invasion of tumor cells (21, 22). Notably, one flavanone – MLo-1302 – previously studied by us in RCC, not only inhibited DNMT3A activity, inducing the re-expression of silenced tumor suppressor genes, but also displayed an anti-neoplastic activity *in vitro* and *in vivo* (23). Therefore, the use of flavonoid compounds might hold therapeutic potential for the treatment of metastatic and resistant RCC.

MLo-1508 is a new 3-bromo-3-nitroflavanone compound, member of the same drug class as MLo-1302, that was found to inhibit DNMT3A activity at low concentrations and with reduced toxicity (24), thus being a promising candidate for RCC treatment.

Hence, in this work, we investigated the effect of MLo-1508 in RCC and explored its combination with the TKI Sunitinib for the treatment of pRCC.

## 2 Materials and methods

### 2.1 Cell lines

In this work, both non-malignant (HKC8) and RCC cell lines (786-O, primary ccRCC; Caki-2, primary pRCC; ACHN, metastatic pRCC) were used (**Supplementary Table 1**). HKC8 is a non-tumorigenic cell line derived from kidney cortex cells, used for the purpose of assessing drug toxicity. While both ccRCC cell lines, 786-O and Caki-2, present distinct origins and transcriptomic profiles. 786-O, derived from a primary lesion, lacks von Hippel-Lindau (*VHL*) expression, while Caki-2 is a VHL-positive cell lines, derived from a cutaneous metastasis and used in the context of metastatic RCC. ACHN, a VHL-positive, tumor protein 53 (*TP53*) mutated cell line, was derived from a pleura metastasis of a papillary renal tumor. The cell lines were culture at 37°C in a 5% CO2 atmosphere with the recommended culture medium (**Supplementary Table 1**) supplemented with 10% fetal bovine serum (FBS; Biochrom, Merck, USA) and 1% penicillin-streptomycin (GRiSP, Portugal). All the cell lines were routinely tested for *Mycoplasma* spp. contamination (TaKaRa PCR Mycoplasma Detection Set, Clontech Laboratories, USA).

### 2.2 Drugs

MLo-1508 [synthetized as in (24)] and Sunitinib (APExBIO, USA) were first dissolved in dimethyl sulfoxide (DMSO; Sigma-Aldrich, Germany) at 10mM and afterwards, intermediate working solutions (1μM – 10μM) were prepared in DMSO and stored at -20°C until further use.

### 2.3 Viability assay

The 3-(4,5-dimethylthiazol-2-yl)-2,5- diphenyltetrazolium-bromide (MTT) assay (Sigma-Aldrich, Germany) and the Resazurin Cell Viability Assay (Canvax Biotech, Spain) were used to assess cell viability upon MLo-1508 and Sunitinib treatment, respectively. For the MTT assay, HKC8 (1.5K cells), 786-O (2K cells), Caki-2 (3K cells) and ACHN (2K cells) were seeded into 96-well plates and treated every 24 hours (h), for 3 days, with different concentrations of MLo-1508 (0μM – 34μM) or the drug vehicle (DMSO). For Sunitinib treatment, the impact on ACHN cell viability was measured using the Resazurin assay. For that, ACHN cells (2K cells) were seeded and treated every 24 h with a broad Sunitinib concentration range (0μM – 10μM) for 72 h. Afterwards, resazurin solution was used to assess cell viability according to the manufacturers’ instructions. Both the absorbance (OD) and the fluorescence intensity, respectively, were measured in the Fluostar Omega microplate reader (BMG Labtech, Germany). In each condition, three technical replicates and at least three biological experiments were performed, and all the values obtained were normalized to the 0 h timepoint.

### 2.4 Apoptosis assay

MLo-1508 effect on apoptosis was assessed using the APOPercentage™ apoptosis assay kit (Biocolor Ltd., Northern Ireland). Briefly, 786-O (30K), Caki-2 (40K) and ACHN (35K) were seeded onto 24-well plates and after 72 h of treatment, the assay was performed according to the manufacturer’s guidelines. The absorbance was determined using the FLUOstar Omega microplate reader (BMG Labtech, Germany) at 550 nm wavelength with background subtraction at 620 nm. Three experimental and three biological replicates were performed. Apoptosis levels were calculated according to the formula:


$$\frac{\text{apoptosis OD}}{\mathrm{mean MTT OD at 72h}}$$

### 2.5 Morphometric assay

After 72 h of MLo-1508 treatment, Olympus CellSens Dimension software (Olympus Corporation, Japan) was used to analyze cell morphometric aspects (area and sphericity). For that, the free-hand polygon tool was applied to, at least, 50 cells on each condition, from three independent experiments.

### 2.6 Single cell gel electrophoresis

To evaluate the DNA fragmentation induced by 3 days of treatment with MLo-1508, 50K cells of each RCC cell line were harvested and re-suspended in low-melting point agarose (Invitrogen, USA), which was then transferred to a microscope slide for polymerization. After lysis and incubation in alkaline electrophoresis buffer for DNA unwinding, single cell gel electrophoresis was performed. Finally, the cells were submerged in neutralization buffer, fixed, and stained with Sybr Green^®^ (Life Technologies, USA). The DNA fragmentation was determined by measuring four previously well described parameters (36). At least 50 cells were measured for each of the three replicates, both in drug and vehicle conditions.

### 2.7 Invasion and migration assays

The effect of MLo-1508 on ACHN cell invasion and migration capabilities was assessed using Falcon^®^ Permeable Support for 24-well plate with 8.0 μm Transparent PET Membrane (Corning, USA) and Nunc^®^ Cell Culture Inserts in 24-well Nunclon Delta surface plate (Sigma-Aldrich, USA), respectively, as previously described (25). For that, after 72 h of MLo-1508 treatment, 15K ACHN cells were harvested and added to the upper chamber in serum-free medium, following the previously described protocol (25). All the inserts were photographed on an Olympus SZX16 stereomicroscope using the Olympus SC180 digital camera (Olympus, Japan). For quantification purposes, five fields within each insert were photographed and the stained cells were counted using the Cell Counter Plugin on ImageJ software. In each condition, three biological experiments were performed, and all the values obtained were normalized to the drug vehicle.

### 2.8 RNA extraction, cDNA synthesis and RT-qPCR

The cell lines’ RNA was extracted using TRIzol (GRiSP, Portugal) and cDNA was synthesized with the RevertAid Reverse Transcription Kit (Thermo Fisher Scientific, USA), following the manufacturer’s instructions. The PCR program was performed in the 7500 Real Time PCR System (Applied Biosystems, USA). Xpert Fast Sybr (GRisP, Portugal) and NZYSpeedy qPCR Probe Master Mix (2X) ROX (NZYTech, Portugal) were used to quantify the target gene expression levels when primers or TaqMan^®^ expression assays (**Supplementary Table 2**) were used, respectively. All the samples were run in triplicates and the expression levels were normalized to *GUSB* (primer or expression assay). Human reference total RNA (Agilent Technologies, USA) was used to generate a standard curve (1:10 series dilutions).

### 2.9 DNA extraction and global DNA methylation analysis

DNA from all the cell lines was extracted by the phenol-chloroform method and 200 ng were used to quantify the global content of 5-methylcytosine (^5^mC) after MLo-1508 treatment. For that, Imprint^®^ Methylated DNA Quantification Kit (Sigma-Aldrich, Germany) was used according to the manufacturer’s recommendations. DNA methylation levels were quantified by measuring the absorbance (450nm) in the FLUOstar Omega microplate reader (BMG Labtech, Germany), and using fully methylated DNA as positive control. In this assay, three independent biological replicates were used.

### 2.10 Quantitative methylation-specific PCR (qMSP)

Sodium bisulfite conversion was performed in 1000 ng of DNA extracted from the MLo-1508 treated cell lines using the EZ DNA Methylation GoldTM Kit (Zymo Research, USA), following the manufacturer’s protocol. All the primers and probes used (**Supplementary Table 3**) were designed using the Methyl Primer express Software v1.0 (Applied Biosystems, USA). In brief, 1 μL of the previously modified DNA and 9 μL of Xpert Fast Probe Master Mix (GRiSP, Portugal) were added to the wells and the reaction was then performed in the 7500 Real Time PCR System (Applied Biosystems, USA). Serial dilutions (1:5) of the bisulfite modified CpGenomeTM Universal Methylated DNA (Sigma Aldrich, Germany) were used to create a calibration curve from which the methylation levels were calculated. In all samples, triplicates were used, normalized to *β-ACTIN* and only amplification cycles ≤ 35 were considered.

### 2.11 DNMT3A activity assay

Following the manufacturer’s protocol, the Nuclear Extract kit (Active Motif, Belgium) was used to obtain nuclear extracts from the treated cell lines, that were quantified by the Pierce BCA Protein Assay kit (Thermo Fisher Scientific, USA). Afterwards, and following manufacturer’s recommendations, DNMT3A activity was measured by applying 10 μg of nuclear extracts to the EpiQuickTM DNMT3A Assay Kit (Epigentek, USA). Absorbance was then measured in the FLUOstar Omega microplate reader (BMG Labtech, Germany) at 450nm and background subtraction at 655nm. Three biological replicates were performed for both vehicle and MLo-1508 drug concentrations.

### 2.12 Synergy assay

ACHN (2K) cells were seeded in 96-well plates and treated every 24 h with a combination of MLo-1508 (0μM – 10μM) and Sunitinib (0μM – 10μM), in a checkerboard system, as previously described (25). The percentage of growth inhibition was measured 72 h after treatment by the Resazurin Cell Viability Assay (Canvax Biotech, Spain), and the combination index (Ci) was calculated using CompuSyn Inc. software (26), as previously reported (25). All experiments were performed with three technical and at least three biological replicates.

### 2.13 Cell cycle analysis

The effect of MLo-1508 and Sunitinib combination on cell proliferation/cell cycle progression of ACHN cells was assessed by flow cytometry using the Phase-Flow™ APC BrdU kit (Biolegend, USA). For that, 50K ACHN cells were harvest after 72 h of ML-1508 and Sunitinib treatment, both alone and in combination, washed with cell staining buffer (Biolegend, USA), and processed according to the manufacturer recommendations. Cells were acquired and analyzed using the FACS Canto™ II Cell Analyzer (BD Bioscience, USA), and FlowJo™ software (BD Biosciences, USA), respectively. Three independent biological replicates were used in the assay.

### 2.14 Chicken chorioallantoic membrane (CAM) assay

The anti-neoplastic effect of MLo-1508 both alone, and in combination with Sunitinib, was assessed *in vivo* using fertilized chicken eggs (Pintobar, Portugal), as previously described (23). At day 10 of development, 2×106 ACHN cells/egg were collected and resuspended in 25μL of Matrigel (BD Biosciences, USA) and implanted into the CAM under sterile conditions. After tumor assembly, eggs were assigned to four treatment groups, including the drug vehicle (DMSO), MLo-1508 (2.06μM), Sunitinib (3.32μM) and drug combination (MLo-1508, 2.06μM and Sunitinib, 3.32μM). CAM implants were pictured both prior and 72 h after in ovo treatment using the Olympus SZX16 stereomicroscope and the digital camera SC-180 (Olympus, Japan). At the end of the experiment, the embryos were sacrificed at – 80°C for 10 min. Both the implants and the underlying CAM portions were cut and fixed in paraformaldehyde solution at 4% (v/v). Ex ovo images were then captured for each condition. The collected membranes were placed into histological cassettes and processed for immunohistochemical (IHC) analysis. H&E staining was performed, and a necrotic tumor score was established by a trained pathologist. Tumor size (perimeter and area) and vascular network were quantified using ImageJ software.

### 2.15 Immunohistochemical analysis

CAM paraffin blocks were cut in 3 μm sections and analyzed by IHC using the Novolink Max Polymer Detection System (Leica Biosystems, Germany), as previously described by us (27). Primary antibodies used in this study are listed in **Supplementary Table 4**. The IHC evaluation was performed by a trained pathologist and the Ki-67 Combined Score was calculated as previously described by Outeiro-Pinho et al. (28). For that, intensity (1 – weak, 2 – moderate, and 3 – strong) was multiplied by the percentage of positive cells (1: 0%-20%, 2: 21%-40%, 3: 41%-60%, 4: 61%-80%, 5: 81%-100%). Representative pictures were taken using Olympus BX41 microscope with Olympus U-TVO.63XC digital camera (Olympus, Japan).

### 2.16 Statistical analysis

To compare the results obtained in each experiment regarding the different drug concentrations and vehicle, Kruskal-Wallis with *post-hoc* Dunn’s multiple comparison test and one-way analysis of variance (ANOVA), with *post-hoc* Dunnet’s multiple comparison test, were used. Additionally, two-way analysis of variance (ANOVA) was performed for the combination assay. The statistical analysis was executed in the GraphPad Prism 7 (San Diego, USA) and statistical significance was achieved when p-value < 0.05.

## 3 Results

### 3.1 MLo-1508 reduces RCC cell viability and promotes apoptosis

**Figure 1:**
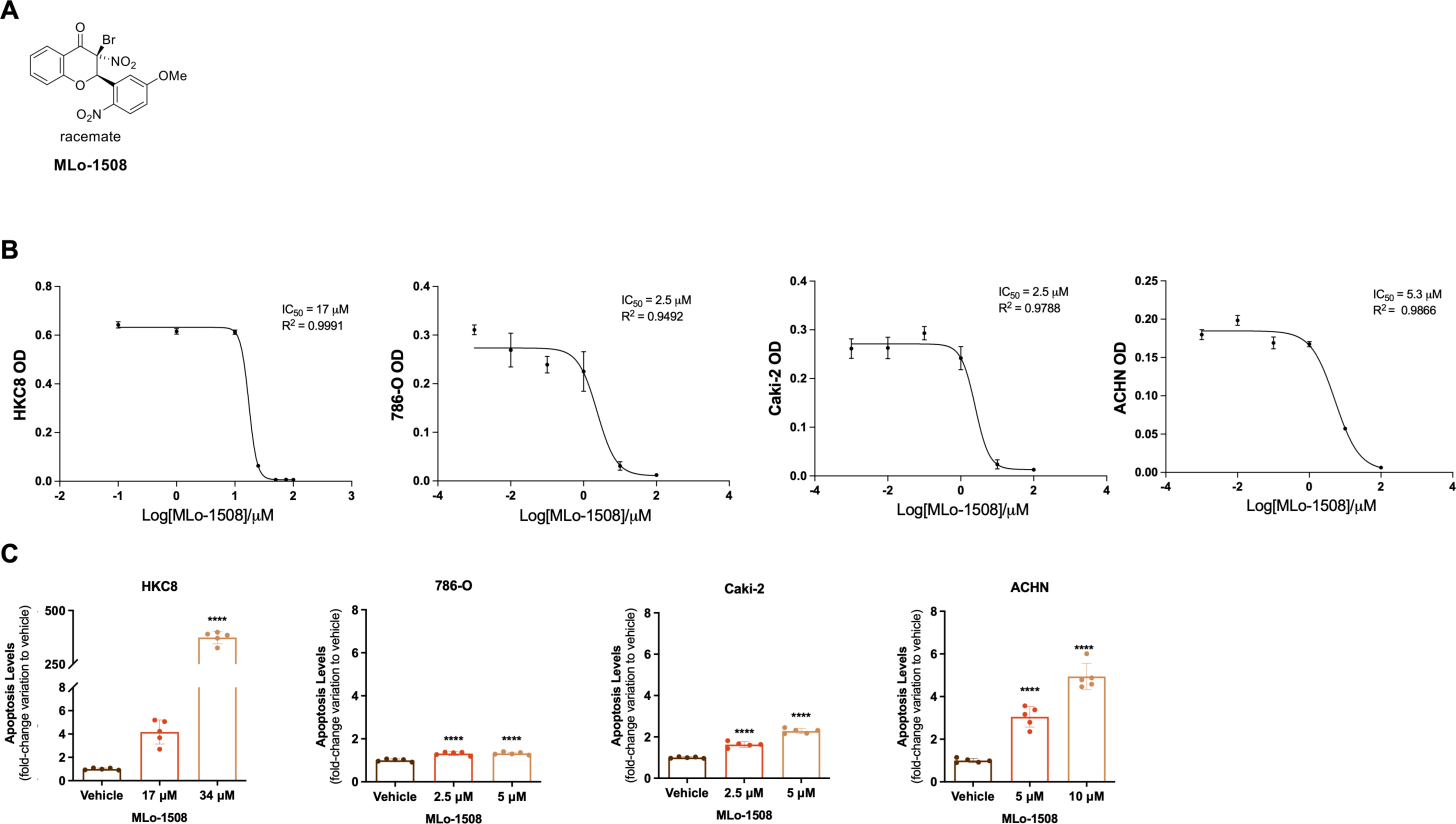
MLo-1508 displays anti-tumoral effects in RCC cell lines. **(A)** MLo-1508 structure. **(B)** MLo-1508 IC_50_ values calculated for HKC8, 786-O, Caki-2 and ACHN. **(C)** MLo-1508 increased apoptosis of RCC cell lines at the IC_50_ and 2xIC_50_ concentrations. The data is presented as mean±SD (n=3). ANOVA with *post-hoc* Dunnet’s multiple comparison test: *p<0.05, **p<0.01, ***p<0.001, ****p<0.0001.

To assess the anti-neoplastic effects of MLo-1508 (**Figure 1A**) in RCC, we started by treating different RCC and a non-malignant cell line for 3 days, with different drug concentrations to determine the effective concentration that decreases cell viability to 50% (IC_50_). Our results showed that MLo-1508 decreased RCC cell viability in a dose-dependent manner (**Figure 1B**, **Supplementary Figure 1A**) at concentrations substantially lower than the one exhibiting cytotoxic effect on the non-malignant HKC8 cell line (**Figure 1B**, **Supplementary Figure 1A, Supplementary Table 5**). Additionally, we observed that the treated ACHN cells displayed a statistically significant decrease in cell viability in both treatment conditions, particularly after 3 days of exposure (**Supplementary Figure 1A**).

We next asked whether these effects were accompanied with apoptotic events. A significantly increase in apoptosis in all RCC cell lines was observed, being the effect more noteworthy in ACHN cells (**Figure 1C**).

These anti-tumoral properties were corroborated by the significant increase in *CASP3* and *CDKN1A*, and the significant decrease in *Ki-67* transcript levels, particularly in ACHN cells (**Supplementary Figure 1B**), suggesting that MLo-1508 might have therapeutic potential in RCC.

### 3.2 MLo-1508 induces morphometric alterations and DNA damage in RCC

**Figure 2:**
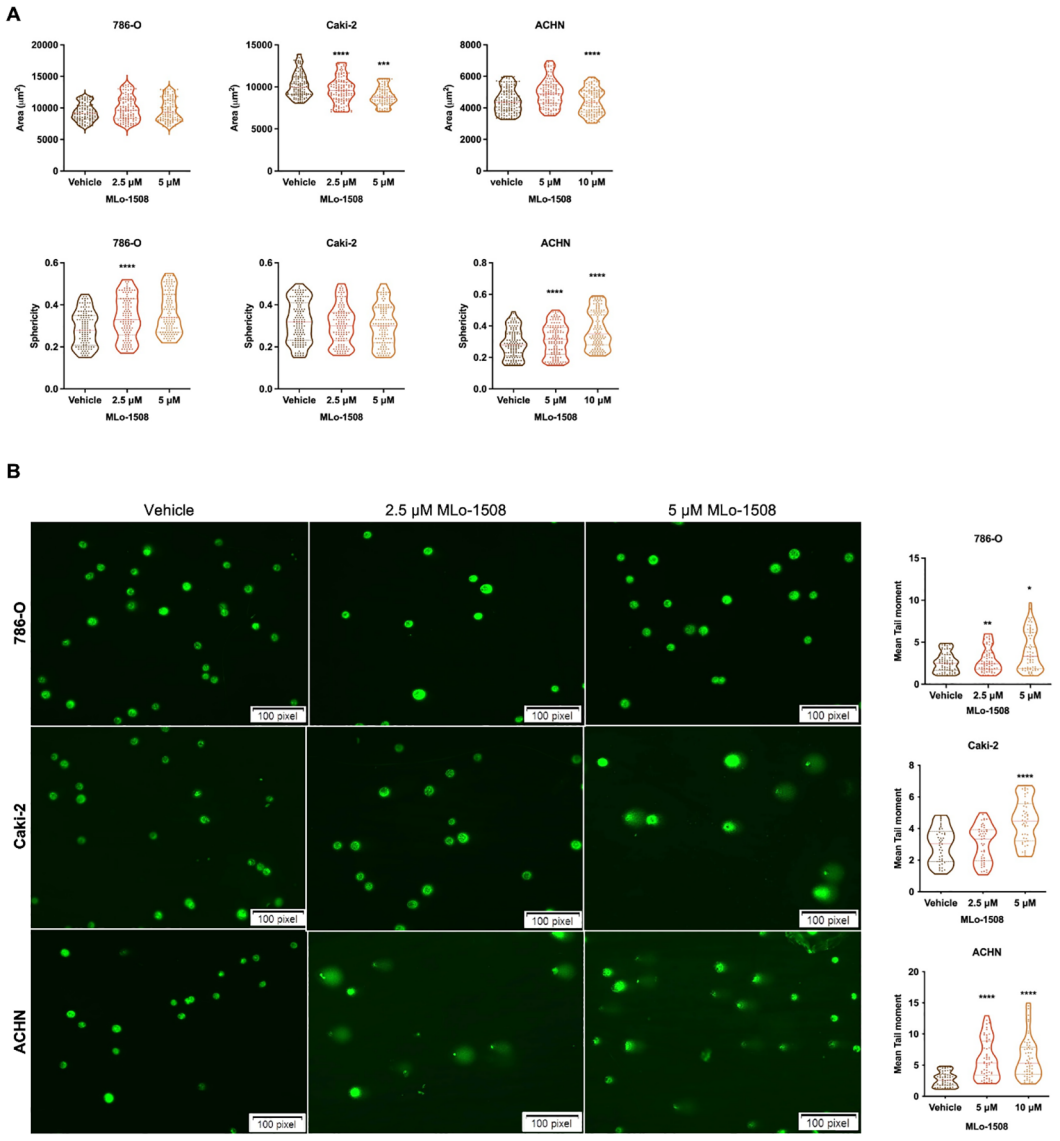
MLo-1508 promotes morphometric alterations and DNA damage in RCC cell lines. **(A)** MLo-1508 altered 786-O, Caki-2 and ACHN cell area (top panel) and sphericity (bottom panel); **(B)** Treatment with MLo-1508 increased RCC cells’ tail moment and length in a dose-dependent manner. The data was analyzed using Comet Assay IV software, with Sybr Green as counterstain. Scale: 100 pixels. All the data are presented as mean±SD (n=3). Kruskal-Wallis test with *post-hoc* Dunn’s multiple comparison test: *p<0.05, **p<0.01, ***p<0.001, ****p<0.0001.

The 3-nitroflavone compounds were previously associated with morphometric alterations and DNA damage, including in RCC (23). Therefore, we further explored the effect of MLo-1508 on cell morphometry and tail length. Overall, after 3 days of treatment, we observed alterations in both cell area and sphericity (**Figure 2A**), which is consistent with the apoptosis observed (**Figure 1C**). Significant alterations in treated 786-O and ACHN cells were depicted, particularly in cell sphericity (**Figure 2A**). Considering that epithelial-mesenchymal transition (EMT) players might have a role in cell sphericity, we assessed the transcript levels of classic players and found a significant decrease in NCAD expression after MLo-1508 treatment (**Supplementary Figure 2A**), although no significant effects were detected in cell invasion (**Supplementary Figure 2B**) and migration capabilities (**Supplementary Figure 2C**).

Additionally, we disclosed that MLo-1508 induced DNA damage in RCC cells, as shown by the significant increase in comet tail movement (**Figure 2B**), and the increase in the DNA damage-related genes *ATR*, *GADD45B* and *RAD9* expression (**Supplementary Figure 2D**). As previously, ACHN was the most responsive cell line to MLo-1508 treatment.

### 3.3 MLo-1508 inhibits DNMT3A activity without reversing the methylation status of RCC-related genes

**Figure 3:**
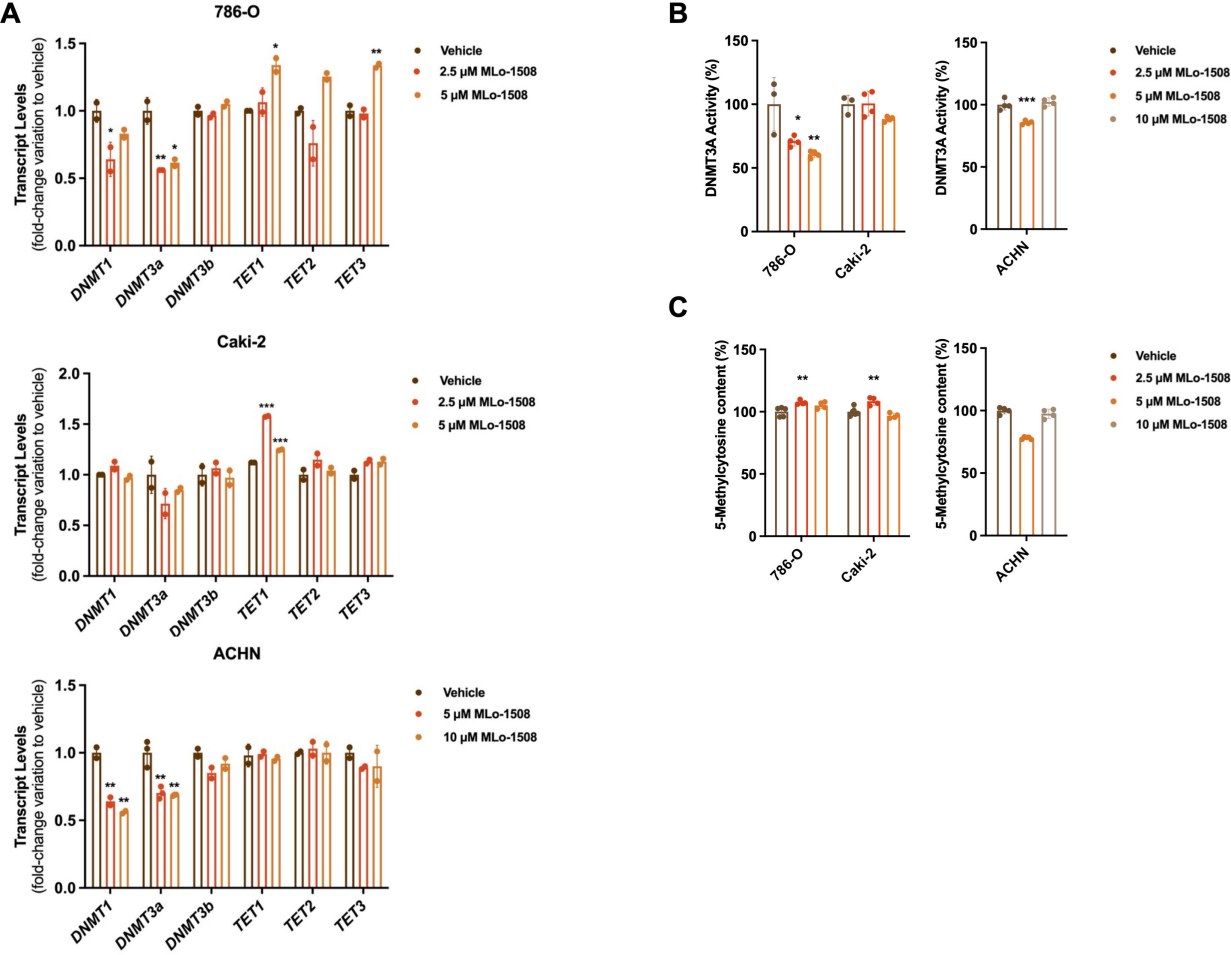
MLo-1508 effect on DNA methylation of RCC cell lines. Effect of MLo-1508 treatment in *DNMT* and *TET* transcript levels **(A)**, DNMT3A activity **(B)** and global DNA methylation levels **(C)**. All the data are presented as mean±SD (n=3). Kruskal-Wallis test with *post-hoc* Dunn’s multiple comparison test: *p<0.05, **p<0.01, ***p<0.001, ****p<0.0001.

Considering the described MLo-1508 epigenetic mechanism of action, we investigated the treatment effect on *DNMT* and *TET* expression levels. We found that MLo-1508 significantly decreased *DNMT1* and *DNMT3A* transcript levels in 786-O and ACHN cells (**Figure 3A**). Moreover, increased *TET1* transcript levels were depicted in Caki-2 and 786-O cells, the latter additionally presenting increased *TET3* expression levels (**Figure 3A**). Additionally, DNMT3A activity was significantly diminished in the treated 786-O and ACHN cells (**Figure 3B**), although the global DNA methylation levels were only significantly impacted in ACHN cells (**Figure 3C**).

Nonetheless, at the gene level, the treatment did not consistently alter the promoter methylation status (**Supplementary Figure 3A**) or transcript levels (**Supplementary Figure 3B**) of RCC-related genes.

### 3.4 Combination of MLo-1508 with Sunitinib demonstrates therapeutic benefit in papillary RCC cells

Following the grander drug response to MLo-1508 detected for pRCC ACHN cells, we then sought to understand if this epi-drug could decrease the effective doses of the standard of care sunitinib, for which a high rating of drug resistance is described between patients (29).

**Figure 4:**
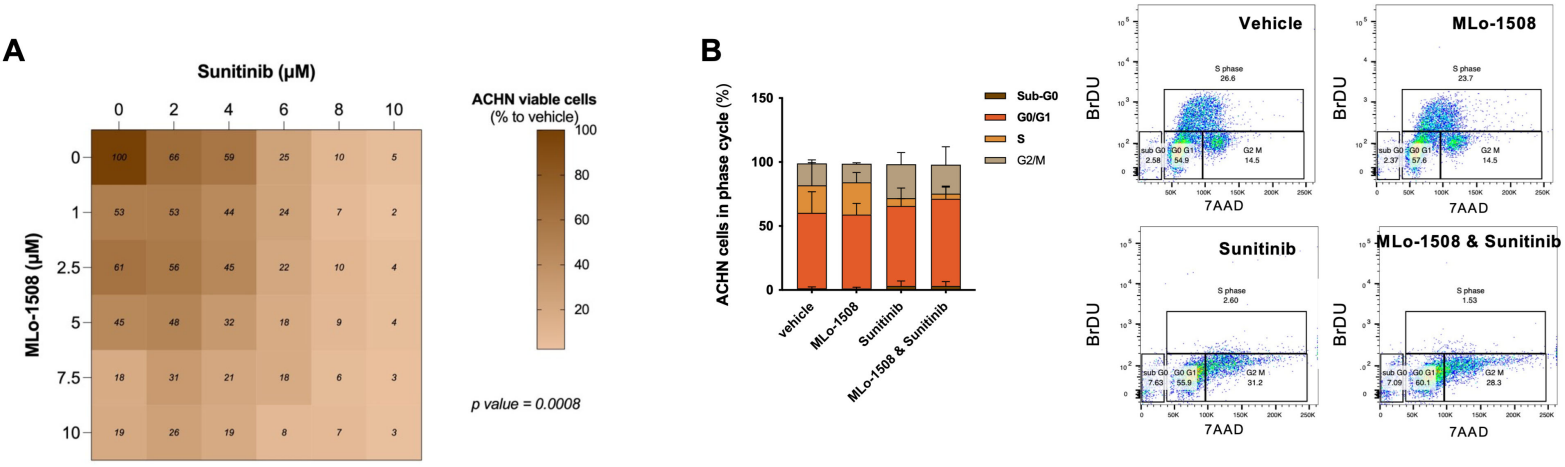
*In vitro* anti-neoplastic effects of combining MLo-1508 and Sunitinib for the treatment of papillary RCC. **(A)** Combination matrix of ACHN cells treated with MLo-1508 and Sunitinib, displaying the percentage of cell viability calculated for each drug. Two-way ANOVA test. **(B)** Effect of the drug combination on ACHN cell cycle progression (left panel) and representative dot plots (right panel). The data is presented as mean±SD of, at least, three biological replicates, each in triplicates. It was used 2.06μM of MLo-1508 and 3.32μM of sunitinib.

As expected, Sunitinib significantly decreased ACHN cell viability (**Supplementary Figures 4A, B**, **Supplementary Table 6**). Importantly, when combined with MLo-1508, a significant dose-dependent reduction in ACHN cell viability was observed (**Figure 4A**), without cytotoxic effects depicted for non-malignant renal cells (**Supplementary Figure 4C**). Notably, the IC_50_ of the drug combination was obtained at drug concentrations inferior to the ones calculated for the single drugs (**Supplementary Table 7**), although without drug synergism (**Supplementary Figure 4D**).

Since sunitinib is a vascular endothelial growth factor (VEGFR) inhibitor, we questioned whether proliferation rates could be diminished by the drug combination. Indeed, MLo-1508 and sunitinib combined treatment led to a decreased percentage of ACHN cells in S-phase, while inducing cell cycle arrest at G2/M-phase, when compared to single treatments (**Figure 4B**).

Hence, MLo-1508 decreased the required sunitinib concentration to produce response, potentially sensitizing pRCC cells to low sunitinib doses, which might aid in delaying resistance acquisition, although without an inherently synergistic function.

### 3.5 MLo-1508 and Sunitinib combinatory effects in an *in vivo* model of pRCC

To further assess the *in vivo* benefit of combining MLo-1508 with sunitinib, a CAM assay was conducted using the responsive pRCC cell line ACHN.

**Figure 5:**
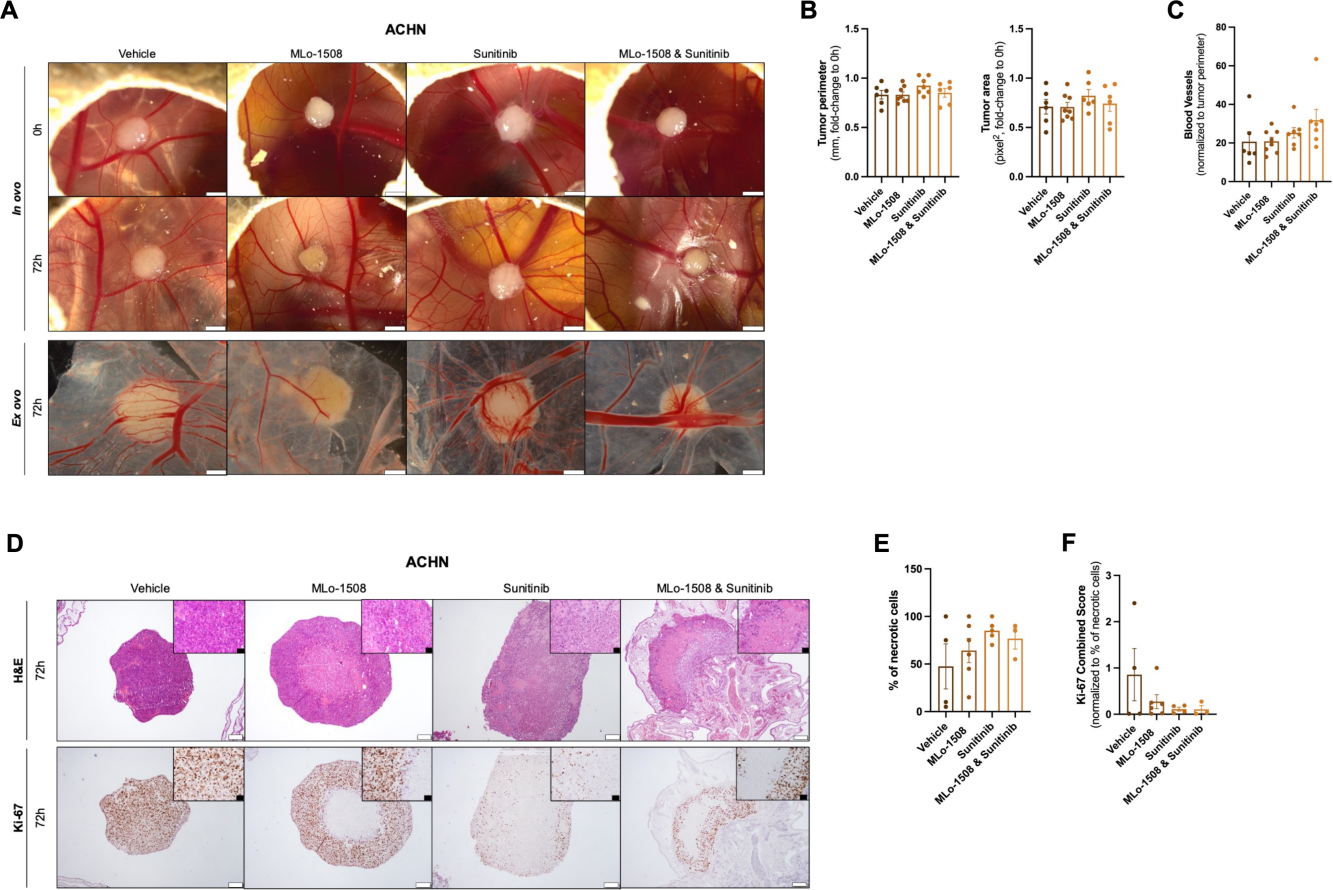
*In vivo* efficacy of MLo-1508 and Sunitinib combination in papillary RCC. The *in vivo* effect of MLo-1508 and Sunitinib combination was assessed using CAM assay **(A–F)**. **(A)** Representative images of ACHN microtumors both *in ovo* and *ex ovo*, before and after drug treatment. Scale bar: 2mm. Drug effect on tumor perimeter and area **(B)**, number of recruited blood vessels **(C)**, tumor burden **(D)**, necrosis **(E)** and Ki-67 expression **(D, F)**. In **(D)**, the white scale bar is of 200μm, and the black scale bar is of 50μm. The data is presented as mean±SD of, at least, six eggs per group. It was used 2.06μM of MLo-1508 and 3.32μM of sunitinib.

After tumor assembling, ACHN microtumors were treated with MLo-1508 and sunitinib, both alone and in combination. After 72 h of treatment, a decrease in tumor 3D size was depicted for all treatment groups (**Figure 5A**), although no differences were observed for tumor perimeter and area measurements, as well as blood vessels recruitment, between vehicle and treated microtumors (**Figures 5A–E**).

Nevertheless, an increased percentage of necrotic cells were depicted in both sunitinib and combination groups (**Figures 5D–F**), highlighting the anti-tumor effect of the combination on cell viability. Additionally, considering the percentage of necrotic cells within each microtumors, we observed a decrease in Ki-67 expression between vehicle and treatment conditions, with the drug combination having the most pronounced effect (**Figures 5D, F**).

## 4 Discussion

Epigenetics, as a hallmark of cancer, has been implicated in cancer onset and progression (8). In RCC, epigenetic mechanisms have been widely described as key players, driving tumor progression and worsening prognosis (10, 12). DNA methylation is the most described epigenetic mechanism in RCC, with DNMT overexpression being associated with worse patient outcomes (30). Hence, targeting this machinery with specific DNMT inhibitors has revealed promising anti-tumoral effects (30). Indeed, several clinical trials testing the effect of DNMT inhibitors, both alone and in combination with the standard of care, are currently ongoing (31–33). Nonetheless, the vast majority of these drugs display reduced effectiveness in solid tumors (34), highlighting the need for the development of new drugs that target DNMT3A and DNMT3B, responsible for *de novo* methylation (11).

Herein, we described, for the first time, the anti-tumoral effects of the DNMT3A inhibitor MLo-1508 in RCC. Remarkably, MLo-1508 reduced RCC cell viability in a dose- and time-dependent manner, while inducing apoptosis at non-toxic concentrations. Moreover, this epi-drug induced alterations in RCC cell morphology, particularly in ACHN cells, in which the IC_50_ concentration increased cell area, that along with the apoptotic levels depicted, might suggest cell death by necrosis (35). The increase in 786-O and ACHN cell sphericity after treatment, which is an epithelial characteristic (36), might explain cell aggressiveness mitigation. Marked DNA damage in all RCC cell lines after treatment was also observed and corroborated by the increase in *ATR*, *GADD45* and *RAD9* transcript levels, suggesting ATR signaling activation (37).

In accordance with the proposed mechanism of action (24), DNMT3A activity was impaired after treatment in 786-O and ACHN cells, along with a reduction in *DNMT1* and *DNMT3A* mRNA levels, although the global content of ^5^mC was only decreased in the pRCC cell line. Indeed, papillary RCC is an aggressive form of the disease presenting a CpG island methylator phenotype (CIMP) (38), that might explain the selectivity of MLo-1508 and the greater anti-neoplastic response observed in this cell line. However, at high doses, particularly in 786-O and ACHN, MLo-1508 seems to restore DNMT1/3A expression and activity, compared to the lower dose, maybe due to compensatory cellular mechanisms. Indeed, some studies have already reported that high doses of DNMT inhibitors, such as azacitidine and decitabine, can restore DNMTs expression and/or activity due to feedback mechanisms that are essential for the cells to maintain DNA methylation levels (39). In particular, high concentrations of decitabine were found to lead to DNMT re-expression after an initial depletion with low doses (39), as observed in this study. Therefore, further studies regarding MLo-1508 dose stratification are required to understand the ideal effective dose, which does not activate compensatory mechanisms.

Nevertheless, no alterations in the gene-specific methylation and mRNA level of RCC silenced genes were detected after treatment, suggesting that MLo-1508 effects on RCC might act beyond DNA methylation. Indeed, the absence of notable demethylation at specific loci indicates that additional epigenetic mechanisms, including histone modifications and non-coding RNAs, might be playing a role in regulating MLo-1508 anti-tumoral effects in RCC. Interestingly, previous studies explored the role of flavone compounds role in regulating chromatin accessibility (39–43). To further clarify the additional epigenetic mechanism involved in RCC-related tumor suppressor gene’s repression, additional molecular studies are needed.

We next sought to understand if MLo-1508 would aid therapeutic benefit to pRCC standard of care. The papillary RCC subtype is treated, as first line, with TKI, namely sunitinib and, recently, cabozantinib (44). However, a numerous percentage of patients treated with sunitinib eventually develop resistance to treatment (29). Thus, we questioned whether the anti-tumoral properties of MLo-1508 would decrease the effective concentration of sunitinib required for treatment, hence delaying resistance acquisition.

As expected, sunitinib effectively decreased ACHN cells viability at an IC_50_ concentration of 3.6μM. When combined with the epi-drug, a significant reduction in ACHN cell viability and cell cycle arrest at G2/M was observed, with no toxicity detected for HKC8 cell lines. Notwithstanding that no drug synergism was depicted, the combination with MLo-1508 diminished sunitinib IC_50_ to 3.3μM.

To further validate these results in a more complex model, a CAM assay was used to assess the drug combination effects *in vivo*. We observed a decrease in the microtumors after 72 h of treatment, although no differences in tumor perimeter, area, or blood vessel recruitment were detected between vehicle and treatment groups. Indeed, the CAM assay allows for the short-term assessment of a drug’s impact on tumor size, which might not entirely mimic the anti-tumoral response in an extended treatment period. To overcome this limitation and further elucidate whether MLo-1508 and Sunitinib combination might impact tumor size, further investigation involving xenograft models would be required to effectively answer this question. Since the ultimate goal of this study was to assess MLo-1508 capability to re-sensitize pRCC to Sunitinib, a decrease in Ki-67 expression and an increase in cell necrosis was observed in the combination-treated microtumors, highlighting the anti-tumoral capability of the drug combination.

Overall, although MLo-1508 exhibited several anti-tumoral properties and its combination with sunitinib seems advantageous as an anti-tumoral strategy, robust synergistic effects are not sustained by CompuSyn data analysis. However, further studies detailing the molecular effects of MLo-1508 can help to unravel novel therapeutic opportunities and new drug combinations that might demonstrate to have stronger anti-neoplastic effects in RCC.

## Data Availability

The original contributions presented in the study are included in the article/**Supplementary Material**. Further inquiries can be directed to the corresponding author.
