# Supplementary material for: Combination of MLo-1508 with sunitinib for the experimental treatment of papillary renal cell carcinoma
Source: Front Oncol. 2025 Mar 24;15:1399956. doi: 10.3389/fonc.2025.1399956 (PMC11973455; doi:10.3389/fonc.2025.1399956)
Supplement: Supplementary file 1 [file DataSheet1.docx]

Supplementary Material

# Supplementary Figures and Tables

## Supplementary Figures

##
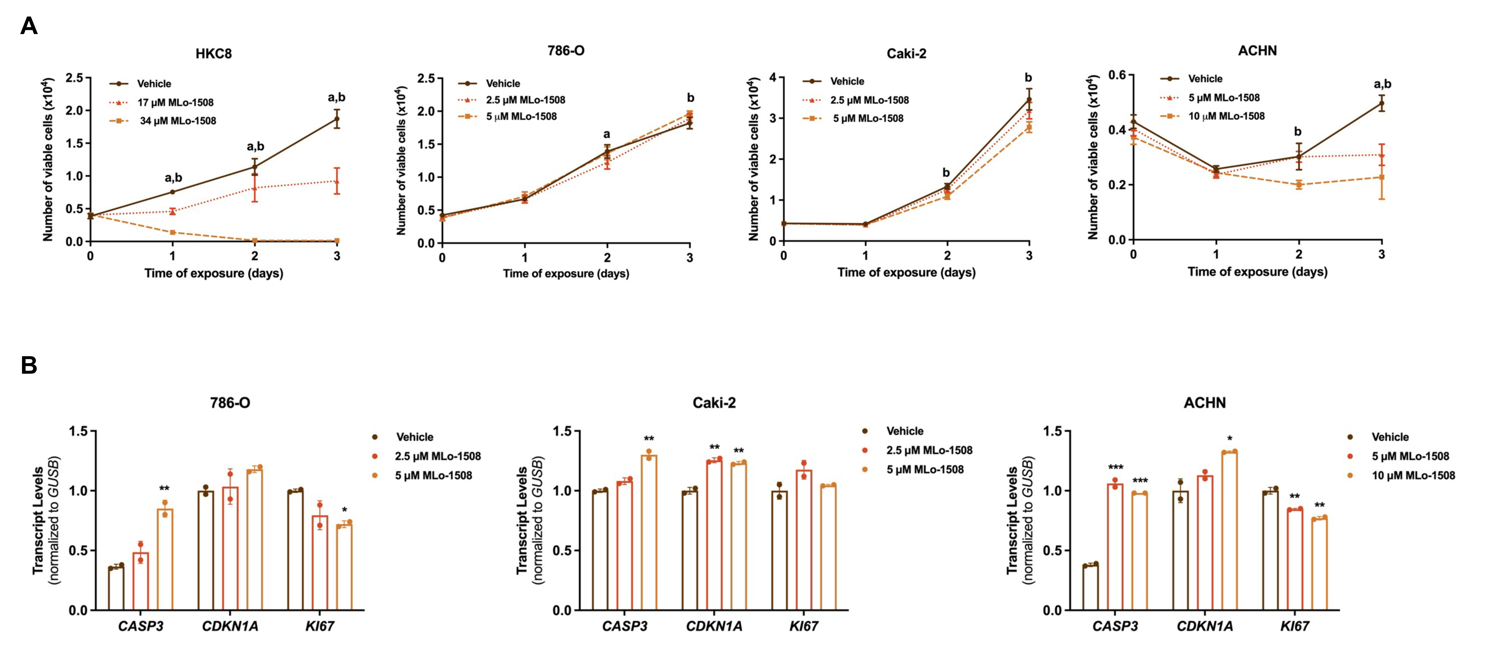
Supplementary Figure 1. (A.) MLo-1508 decreases RCC cell lines viability after 3 days of treatment. The data is presented as mean±SD (n=3). ANOVA with post-hoc Dunnet’s multiple comparison test. a) represents a statistically significant difference between vehicle and IC_50_ conditions, b) represents a statistically significant difference between vehicle and 2x IC_50_ conditions. (B.) MLo-1508 treatment modulates *CASP3*, *CDKN1A* and *Ki-67* transcript levels. All the data are presented as mean±SD (n=3). 1 way ANOVA test with post-hoc Dunnet’s multiple comparison test **p<0.05, **p<0.01, ***p<0.001*.

**
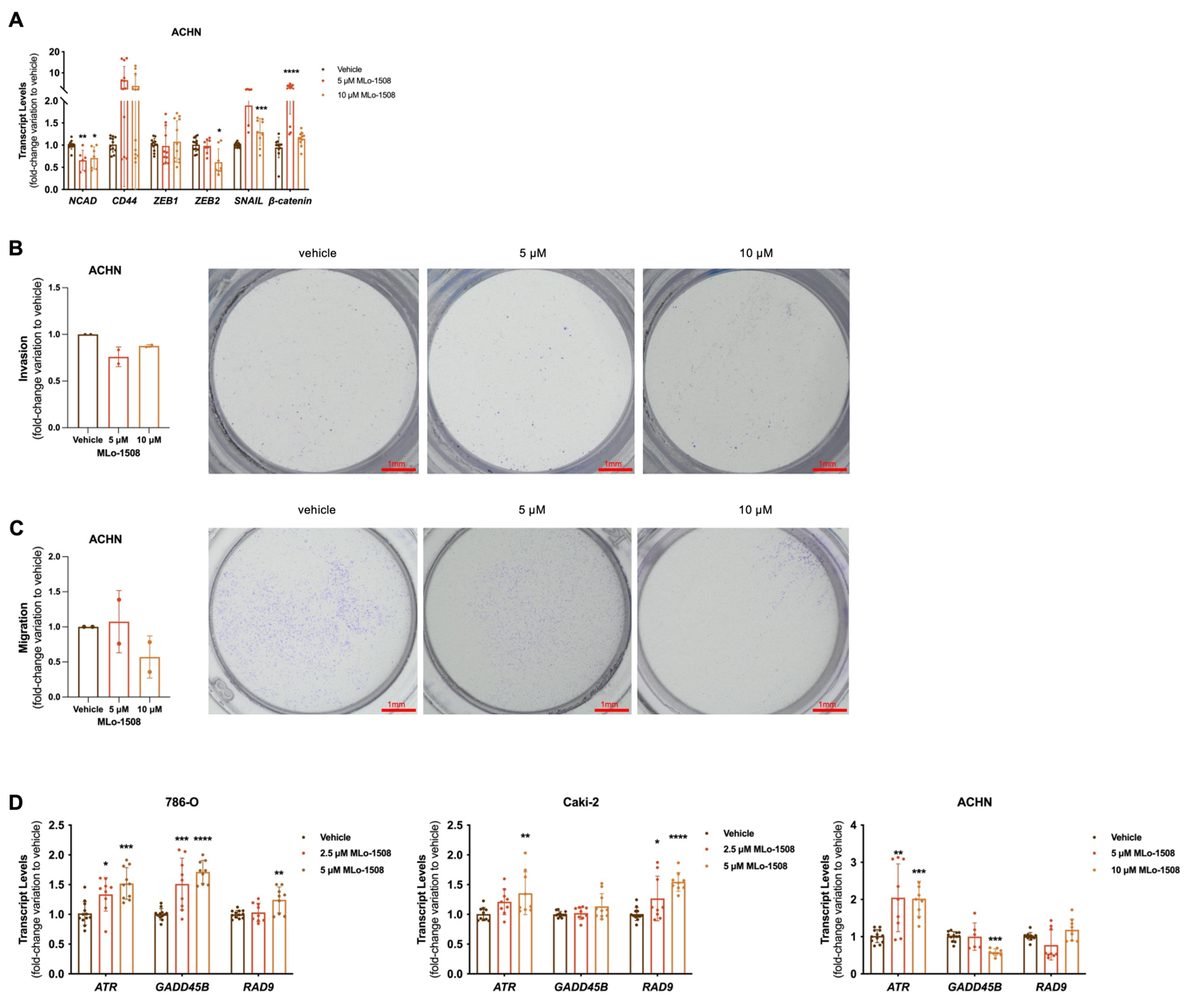
**

**Supplementary Figure 2.** **MLo-1508 effect on RCC aggressive capabilities.** MLo-1508 treatment effect on (**A.**) EMT players expression, and (**B.**) invasion and (**C.**) migration capabilities of ACHN cells. In **B.** and **C.** ACHN cells were stained with Crystal Violet (right panels) and the representative images were taken using the Olympus S2X16 microscope and the digital camera Olympus SC180 (Olympus, Japan). Scale bar: 1mm. **D.** Epi-drug effect on the transcript levels of *ATR*, *GADD45B* and *RAD9* on 786-O, Caki-2 and ACHN. All the data are presented as mean±SD (n=3). Kruskal-Wallis test with post-hoc Dunn’s multiple comparison: **p<0.05, **p<0.01, ***p<0.001, ****p<0.0001.*

**
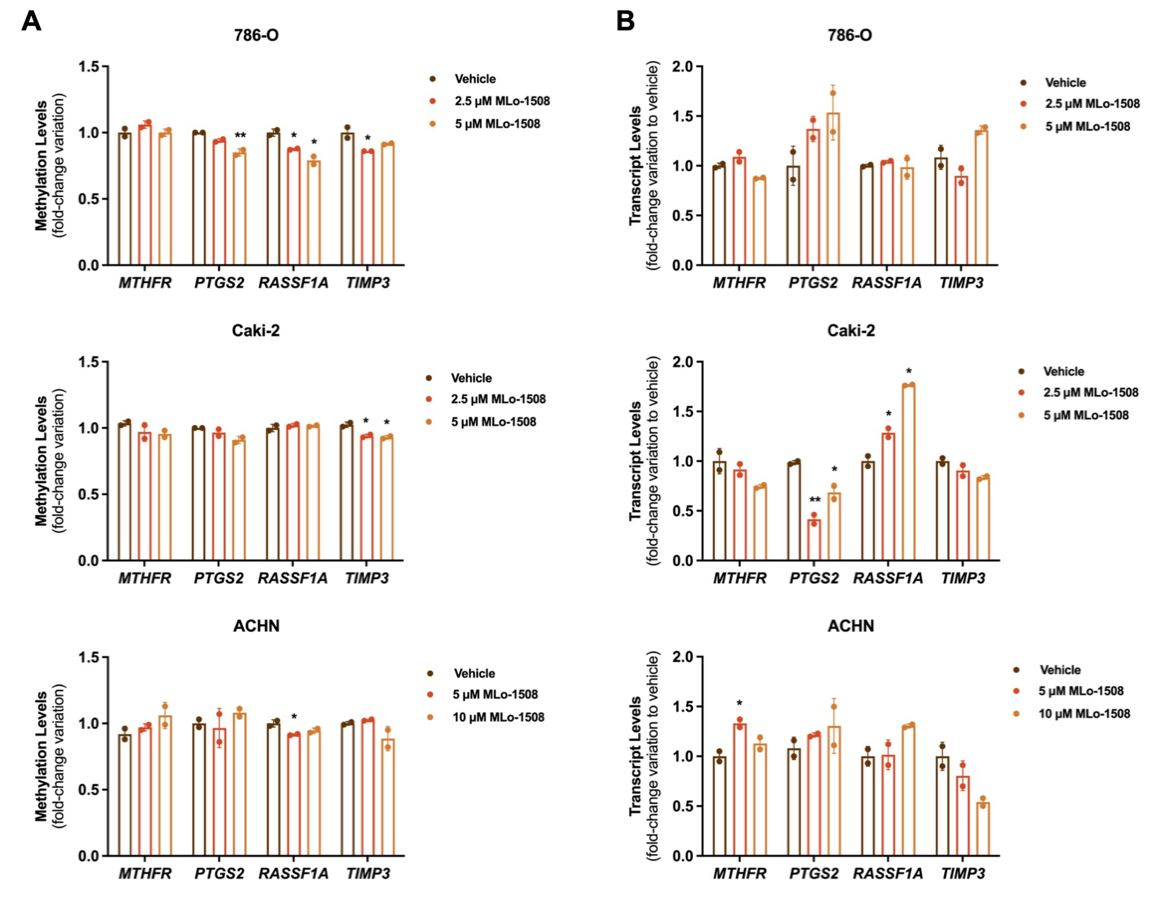
**

**Supplementary Figure 3.** **MLo-1508 effect on RCC-related genes methylation and expression levels.** MLo-1508 impact on the **(A.)** methylation status and **(B.)** transcript levels of *MTHFR*, *PTGS2*, *RASSF1A,* and *TIMP3*. All the values were normalized to **(A.)** *ACTB* and **(B.)** *GUSB*. All the data are presented as mean±SD (n=3). Kruskal-Wallis test with post-hoc Dunn’s multiple comparison: **p<0.05, **p<0.01.*


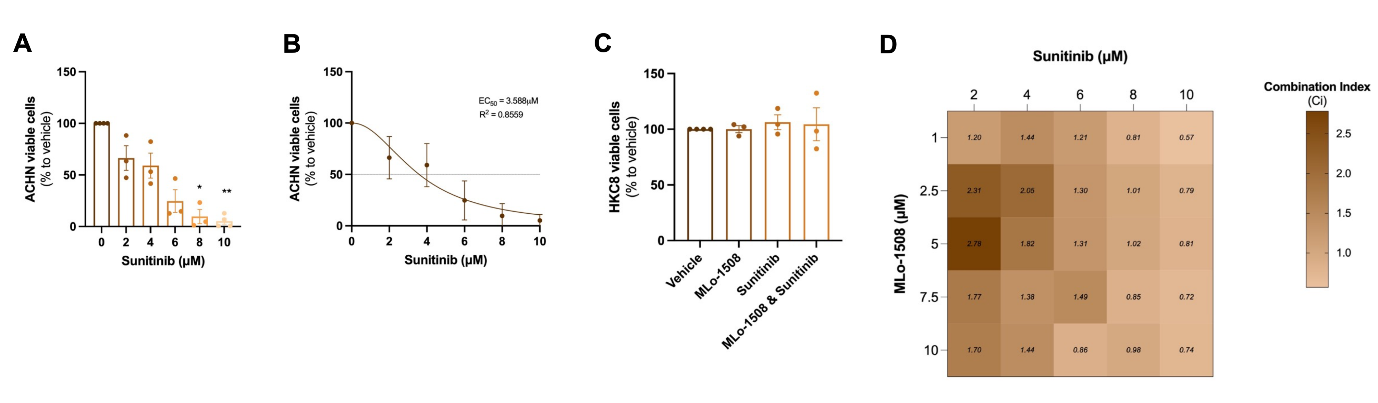


**Supplementary Figure 4.** **MLo-1508 and Sunitinib effects on ACHN cells, *in vitro*. A.** Percentage of viable ACHN cells after 72 h of Sunitinib treatment. **B.** Effect of increasing Sunitinib doses in the percentage of viable ACHN cells and respective IC50 value. **C**. Cytotoxic effect of MLo-1508 and Sunitinib, both alone and in combination, in the non-malignant HKC8 cell line. In **A.-C.** the results are representative of, at least, three independent experiments, each in triplicates, and are presented as percentage to vehicle, mean±SD. Kruskal-Wallis test: **p<0.05, **p<0.01.* **D.** Combination Index (Ci) of MLo-1508 and sunitinib combined treatments, calculated using the CompuSyn software. In **C.** and **D.,** it was used 2.06μM of MLo-1508 and 3.32μM of sunitinib.

**1.2. Supplementary Tables**

**Supplementary Table 1.** Cell lines used in this research.

| **Cell line** | **Provider** | **RRID** | **Growth medium** |
| --- | --- | --- | --- |
| **HKC8** | ATCC^®^ PCS-400-010™ | CVCL_Y910 | RPMI-1640 Medium (Biotecnómica, Portugal) |
| **786-O** | ATCC^®^ CRL-1932™ | CVCL_1051 | RPMI-1640 Medium (Biotecnómica, Portugal) |
| **Caki-2** | ATCC^®^ HTB-47™ | CVCL_0235 | McCoy's 5A (Modified) Medium (Biotecnómica, Portugal) |
| **ACHN** | ATCC^®^ CRL-1611™ | CVCL_1067 | Minimum Essential Medium (Biotecnómica, Portugal) |

**Supplementary Table 2.** Primers sequences, TaqMan expression assays and conditions used in RTq-PCR.

| **Gene** | **Primers Sequence (5’ 🡪 3’)** | **Volume (µL, F+R, 10µM)** | **Annealing Temperature (ºC)** |
| --- | --- | --- | --- |
| ***DNMT1*** | **F:** TATCCGAGGAGGGCTACCTG  **R:** ATGAGCACCGTTCTCCAAGG | 0.5 | 60 |
| ***DNMT3a*** | **F:** TATTGATGAGCGCACAAGA **R:** GGGTGTTCCAGGGTAACATTGAG | 0.5 | 60 |
| ***DNMT3b*** | **F:** GAATTACTCACGCCCCAAGGA **R:** ACCGTGAGATGTCCCTCTTGTC | 0.5 | 60 |
| ***TET1*** | **F:** ACCTGCAGCTGTCTTGATCG **R:** TTTCCCTGACAGCAGCAACA | 0.2 | 62 |
| ***TET2*** | **F:** ACGCTTGGAAGCAGGAGAT **R:** AAGGCTGCCCTCTAGTTGAA | 0.3 | 64 |
| ***TET3*** | **F:** CCCACAAGGACCAGCATAAC **R:** CCATCTTGTACAGGGGGAGA | 0.3 | 62 |
| ***CDKN1A*** | Hs00355782 | 0.5 | 60 |
| ***Ki-67*** | Hs01032427 | 0.5 | 60 |
| ***CASP3*** | Hs00234387 | 0.5 | 60 |
| ***ATR*** | **F:** TAGGGGAATTGGGGGCGATA **R:** TGAATCTTCTACTCCAGTCACAAA | 0.5 | 60 |
| ***GADD45B*** | **F:** CTGGTCACGAACCCTCACAC **R:** CTTTCTTCGCAGTAGCTGGC | 0.5 | 60 |
| ***RAD9*** | **F:** GTGAAGGTGCTCGGCAAGG **R:** CCAAGGGTTCCAGGTAGAGC | 0.5 | 60 |
| ***MTHFR*** | **F:** GGCCTGAAGAACATCATGGCGCTGC **R:** TTCGGATGTGCTTCACCAGG | 0.3 | 60 |
| ***PTGS2*** | **F:** GTCACCATCTCCTTTCTTGA. **R:** CTGATGCGTGAAGTGCTG | 0.5 | 60 |
| ***RASSF1A*** | **F:** AGCGCCCAAAGCCAGCGAAGCACGG **R:** CCCGCAACAGTCCAGGCAGACGAGC | 0.5 | 60 |
| ***TIMP3*** | **F:** TTCGGTTACCCTGGCTACCA **R:** CTGCAGTAGCCGCCCTTCT | 0.3 | 62 |
| ***NCAD*** | **F:** AGGCTTCTGGTGAAATCGCA **R:** GCAGTTGCTAAACTTCACATTGAG | 0.25 | 64 |
| ***CD44*** | **F:** TGGTTGCTTCAAGGACACAT  **R:** GCAAATGCTCTGTTGCAGTG | 0.3 | 60 |
| ***ZEB1*** | **F:** TCCATGCTTAAGAGCGCTAGCT **R:** ACCGTAGTTGAGTAGGTGTATGCCA | 0.15 | 60 |
| ***ZEB2*** | **F:** CGCTTGACATCACTGAAGGA **R:** CTTGCCACACTCTGTGCATT | 0.2 | 60 |
| ***BCatenin*** | **F:** AGGGCTTACTGGCCATCTTT **R:**AAGGTTGTGGAGAGTTGTTAATGG | 0.5 | 60 |
| ***SNAIL*** | **F:** CCCAATCGGAAGCCTAACTA **R:** GGACAGAGTCCCAGATGAGC | 0.25 | 64 |
| ***GUSB*** | **F:** CTCATTTGGAATTTTGCCGATT **R:** CCG AGT GAA GAT CCC CTT T | 0.5 | 61 |
| ***GUSB*** | Hs99999908 | 0.5 | 60 |

F: foward primer; R: reverse primer

**Supplementary Table 3.** Primers, probes sequences, and conditions used in qMSP.

| **Gene** | **Primers Sequence (5’ 🡪 3’)** | **Volume (µL, F+R, 10µM)** | **Annealing Temperature (ºC)** |
| --- | --- | --- | --- |
| ***MTHFR*** | **F:** TGGTAGTGAGAGTTTTAAAGATAGTTCGA **R:** CGCCTCATCTTCTCCCGA  **Probe:** FAM – TCTCATACCGCTCAAAATCCAAACCCG - TAMRA | **F+R**: 0.8  **Probe**: 0.2 | 60 |
| ***PTGS2*** | **F:** CGGAAGCGTTCGGGTAAAG  **R:** AATTCCACCGCCCCAAAC  **Probe:** FAM – TTTCCGCCAAATATCTTTTCTTCTTCGCA - TAMRA | **F+R**: 0.8  **Probe**: 0.2 | 60 |
| ***RASSF1A*** | **F:** GCGTTGAAGTCGGGGTTCG **R:** CCCGTACTTCGCTAACTTTAAACG  **Probe:** FAM - ACAAACGCGAACCGAACGAAACCA -TAMRA | **F+R**: 0.6  **Probe**: 0.2 | 62 |
| ***TIMP3*** | **F:** GCGTCGGAGGTTAAGGTTGTT **R:** CTCTCCAAAATTACCGTACGCG  **Probe:** FAM - AACTCGCTCGCCCGCCGAA - TAMRA | **F+R**: 0.4  **Probe**: 0.2 | 60 |
| ***β-ACTB*** | **F:** TGGTGATGGAGGAGGTTTAGTAAGT **R:** ACCAATAAAACCTACTCCTCCCTTAA  **Probe:** FAM - ACCACCACCCAACACACAATAACAAACACA - TAMRA | **F+R**: 0.4  **Probe**: 0.2 | 60 |

F: foward primer; R: reverse primer

**Supplementary Table 4.** Primary antibodies used in the Immunohistochemistry analysis.

| **Antibody** | **Company** | **Catalogue Number** | **Positive Control** | **Antigen Retrieval** | **Antibody Dilution** |
| --- | --- | --- | --- | --- | --- |
| **Ki-67** | Agilent Dako, USA | M724001-2 | Lymphoma | Citrate Buffer (10mM, pH=6)  MW, 20 min | 1:150, ON, RT |

MW – microwave; ON – overnight; RT – room temperature

**Supplementary Table 5.** MLo-1508 effective concentration that decreases cell viability to 50% (IC50) after 3 days of treatment.

| **Cell Line** | **IC_50_ MLo-1508 (µM)** |
| --- | --- |
| **HKC8** | 17 |
| **786-0** | 2.5 |
| **Caki-2** | 2.5 |
| **ACHN** | 5 |

**Supplementary Table 6.** Sunitinib effective concentration that decreases cell viability to 50% (IC50) after 3 days of treatment.

| **Cell Line** | **IC_50_ Sunitinib (µM)** |
| --- | --- |
| **ACHN** | 3.6 |

**Supplementary Table 7.** MLo-1508 and Sunitinib combination that decreases cell viability to 50% (IC50) after 3 days of treatment. CompuSyn Inc. data calculation.

| **Cell Line** | **IC_50_ MLo-1508 (µM)** | **IC_50_ Sunitinib (µM)** |
| --- | --- | --- |
| **ACHN** | 2.06 | 3.32 |
